# Effect of Monomer Dosing Rate in the Preparation of Mesoporous Polystyrene Nanoparticles by Semicontinuous Heterophase Polymerization

**DOI:** 10.3390/molecules20010052

**Published:** 2014-12-23

**Authors:** Dalia Y. Sosa, Lourdes Guillén, Hened Saade, Eduardo Mendizábal, Jorge E. Puig, Raúl G. López

**Affiliations:** 1 Departamento de Procesos de Polimerización, Centro de Investigación en Química Aplicada, Blvd. Enrique Reyna No. 140, C. P. 25294 Saltillo, Coahuila, Mexico; 2 Departamentos de Química e Ingeniería Química, Centro Universitario de Ciencias Exactas e Ingenierías, Universidad de Guadalajara, Blvd. M. García-Barragán No. 1451, C. P. 44430 Guadalajara, Jalisco, Mexico

**Keywords:** mesoporous polystyrene nanoparticles, semicontinuous heterophase polymerization, monomer dosing rate effect

## Abstract

The semicontinuous heterophase polymerization of styrene in the presence of cross-linking and porogen agents was carried out. Latexes with close to 20% solid content, which contained mesoporous nanoparticles with 28 nm in average diameters, up to 0.5 cm^3^/g in porosity and 6–8 nm in pore diameters were obtained. By varying the monomer dosing rate over the micellar solution, an unexpected direct dependence of instantaneous conversion on the monomer dosing rate was found. This was ascribed to the higher average number of radicals per particle attained in the polymerization at the higher dosing rate, which in turn would arise from the higher gel percentage in the polymer. It is believed that the cross-linked chains prevent encounters between radicals, delaying the bimolecular termination reactions and allowing the existence of more than one radical inside the particles, which in turn increases the propagation rate.

## 1. Introduction

The preparation and characterization of porous micro- and nanopolymeric particles is a very attractive and longstanding research topic in view of the actual and potential applications of such particles. Well-established techniques, mainly based on emulsion and suspension polymerization, allow one to prepare in a controlled manner meso- and microporous particles with porosities higher than 1 cm^3^/g, surface areas in the hundreds of square meters and average diameters ranging from some tenths of micrometers to millimeters [1,2,3,4,5,6]. Recently, this kind of structures has become interesting in the development of drug delivery systems [7,8], catalysts [9,10], enzyme and cell immobilization [11], among others. A common feature of these particles is that their sizes are typically higher than 100 nm in average diameter.

Research on mesoporous polymeric nanoparticles with average diameters smaller than 50 nm has received little attention [12,13,14]. The interesting thing about this kind of nanoparticles is their enhanced area/volume ratio and the increased capacity for avoiding the immunological system, a feature especially useful for drug delivery systems due to the fact that it leads to a longer circulation period in the bloodstream [15]. The reports in the specialized literature on the preparation of such small nanoparticles include the work of Lee and Kim [12], who prepared mesoporous polymeric nanoparticles with average diameters smaller than 50 nm and pores of 2–6 nm in diameter by a sol-emulsion-gel method. More recently, Wu *et al.* [14] documented the preparation of porous hyperbranched conjugated polymeric nanoparticles with diameters ranging 20–60 nm by miniemulsion polymerization. On the other hand, our group has reported the preparation of mesoporous polymeric nanoparticles by polymerizing styrene in the presence of cross-linking and porogen agents using the so called semicontinuous heterophase polymerization technique [13]. The obtained nanoparticles showed average diameters close to 30 nm, pore diameters in the range 6–8 nm and porosities around 0.40 cm^3^/g.

Semicontinuous heterophase polymerization is a technique developed by our group [16,17,18] that allows one to obtain polymeric nanoparticles with diameters smaller than 50 nm, solids contents up to 25%–30% and all the surfactant in the latex stabilizing the particles. However, this technique requires one to operate under the so-called *monomer-starved conditions* during the addition period [19]. Krackeler and Naidus [19] coined this term to explain the smaller particle sizes obtained in the emulsion polymerization of styrene carried out in semicontinuous mode compared to the batch process. These authors resorted to the correlation developed for emulsion polymerization by Smith and Ewart [20] for predicting the number of particles (*N*_P_) for the case-II kinetics, in which *N*_P_ is inversely proportional to the volume growth rate of polymer particles during the nucleation period. When the particles are saturated with monomer, they grow at their maximum rate. As a consequence the particle nucleation is minimum. In semicontinuous emulsion polymerization particle monomer saturation is attained by operating at the so-called *monomer-flooded conditions*. In contrast the operation under monomer-starved conditions, that is to say, when monomer in the particles is below the saturation concentration, slows down the particle growth rate resulting in a larger number of smaller particles. These conditions are achieved by adding the monomer at very slow dosing rates.

As described above, semicontinuous heterophase polymerization operating under monomer starved conditions allows the preparation of mesoporous polymeric nanoparticles having average diameters smaller than 50 nm [13]. As far as we know, ours is the only report on the use of this technique for preparing such particles. With the aim to contribute to the understanding of the mentioned polymerization technique when applied to the preparation of ultrafine mesoporous polymeric nanoparticles, we present here the results of a study on the effect of monomer (styrene and divinylbenzene) dosing rate. This variable was chosen due to the fact it is the one that mainly determines the polymerization kinetics and particle size [21], therefore it would be interesting to investigate how the presence of the cross-linking (divinylbenzene) and porogen (toluene) agents affect the known effect of monomer dosing rate.

## 2. Results and Discussion

### 2.1. Polymerization Kinetics

**Figure 1:**
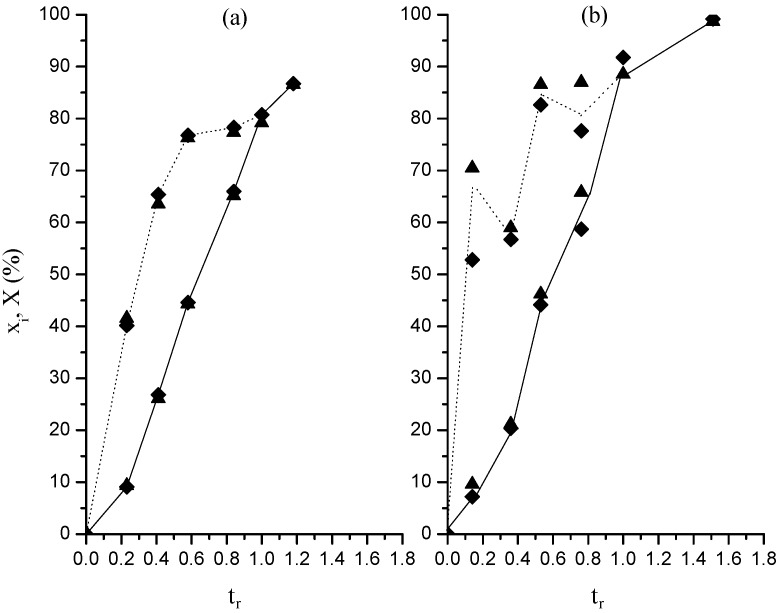
Evolution of instantaneous, *x_i_* (----) and global, *X* (―) conversions with relative time in polymerizations at 0.05 (**a**) and 0.15 (**b**) g/min in organic phase dosing rate. Runs M (♦); runs MR (▲).

The kinetics of the polymerizations carried out in this study are shown in Figure 1. This figure describes the evolution of instantaneous (*x_i_*) and global conversions (*X*) as a function of the relative time (*t_r_*). Here, *x*_i_ is the fraction of added monomer up to time *t* that has changed into polymer; *X*, the fraction of monomer (compared to the total amount to be added) converted to polymer at time *t*, whereas *t_r_* is the ratio of a given time *t* divided by the total addition time. The values of *x_i_* and *X* as a function of *t*, and consequently, of *t_r_* were calculated by the gravimetric method and using the following equations, obtained from a simple mass balances:

(1) $$x_{i}(t)=\frac{w_{pol}}{tF_{0}X_{m}}$$

(2) $$X\left(t\right)=\frac{tF_{0}x_{i}}{M_{t}}$$

where *w_pol_* is the amount of polymer produced up to time *t*, *F_o_* is the organic phase dosing rate, *X_m_* is the monomer concentration in the organic phase and *M_t_* is the total weight of monomers to be added.

In Figure 1 and in the following figures, (a) and (b) correspond to the results of lower and higher organic-phase dosing rate, respectively. Given that in this case toluene is an inert component, it would be also appropriated to say lower and higher monomer dosing rate or simply lower and higher dosing rate hereinafter. The behavior of the *x_i_* curves in Figure 1 is that typically observed in a polymerization carried out in presence of a surfactant at contents higher than its critical micellar concentration by adding the monomer in a semicontinuous fashion to operate under monomer starved conditions [16,17,18]. In a typical behavior *x_i_* values are relatively low during the first stages of polymerization before rising with the reaction time, attaining values higher than 90%–95% under highly monomer starved conditions. In our case it should be noted that the higher *x_i_* values correspond to the polymerization carried out at the higher value of *F_o_*. Taking into account that the only difference between the two polymerizations is *F_o_*, this result contravenes the expected behavior in polymerizations performed under monomer starved conditions. In accordance with the literature the inverse relationship between *x_i_* and monomer dosing rate, while maintaining unchanged the rest of variables, is the very well-known fact [16,17,18,21]. When the monomer dosing rate decreases the monomer concentration inside particles decreases, which leads to a reduction in the propagation rate, resulting in a diminution in the particle volumetric growth. The result is an increase in the particle number density (*N_p_*). The increase in the number of places in which the monomer consumption is carried out, that is to say, the particles, overwhelms the drop in the monomer concentration in the particles, leading to an increase in *x_i_*. The use of a porogen and a crosslinking agent in this study could be the origin of the unexpected relationship between *x_i_* and *F_o_*. In the following, an explanation for this unexpected finding will be provided.

### 2.2. Particle Size and Number Density of Particles

**Figure 2:**
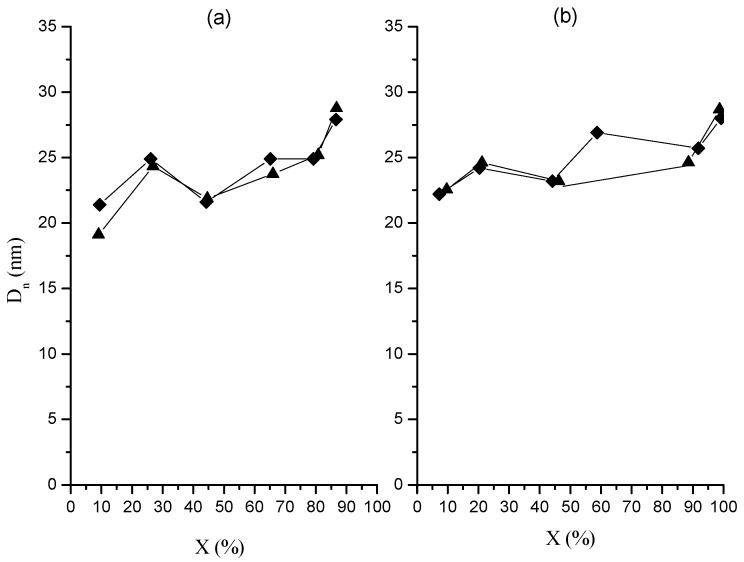
Number average diameter measured by STEM as a function of global conversion in polymerizations at *F_o_* 0.05 (**a**) and 0.15 (**b**) g/min. Runs M (♦); runs MR (▲).

**Figure 3:**
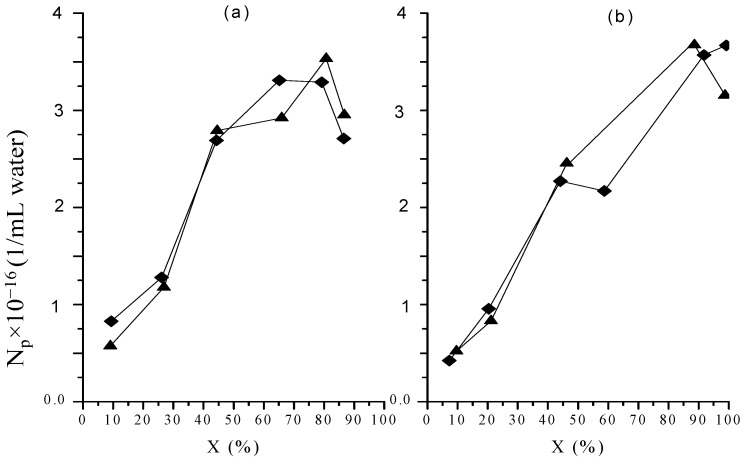
Particle number density as a function of global conversion in polymerizations at 0.05 (**a**) and 0.15 (**b**) g/min in organic dosing rate. Runs M (♦); runs MR (▲).

**Figure 4:**
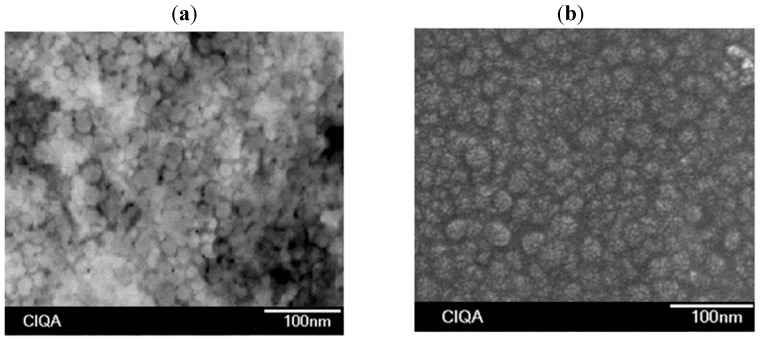
Representative micrographs from samples of final latexes in polymerizations at 0.05 g/min (**a**); 0.15 g/min (**b**) in organic phase dosing rate.

Figure 2 and Figure 3 show the change of number-average particle diameter (*D_n_*) and *N_p_* with *X*, respectively. Figure 4 includes micrographs from final latexes prepared using both dosing rates. To estimate *N_p_* the following equation was used:

(3) $$N_{p}=\frac{6C_{p}}{\pi \rho_{part}D_{n}^{3}}$$

where *C_p_* is polymer concentration in g/mL water and *ρ_par_*_t_ is the density of porous particle in g/mL. This density was calculated using the following equation, developed by considering 1 gram hypothetical porous particle (*P*) with porosity (ϕ) in cm^3^/g:

(4) $$\rho_{part}=\frac{P}{P\phi +\frac{P}{\rho_{p}}}=\frac{1}{\phi +\frac{1}{\rho_{p}}}$$

In this equation ρ*_p_* is the polymer density, for which all the polymer is considered as polystyrene with a density of 1.05 g/mL [22]. Figure 2 depicts a very similar *D_n_* behavior for both polymerizations, with slightly larger sizes for the polymerization carried out at the higher dosing rate up to 60%–65% global conversion. However, the final *D_n_* value was close to 28 nm in both cases. The absence of dependence of final particle size on monomer dosing rate in this study contrasts with the known direct relationship between both these variables in semicontinuous heterophase polymerization under monomer starved conditions [16,17,18,21].

The evolution of *N_p_* shown in Figure 3 is also similar for both polymerizations, with higher values up to 60%–66% global conversion for the polymerizations carried out at the lower dosing rate, though the difference between both polymerizations is more pronounced than that in Figure 2 as a consequence of the dependence of *N_p_* on *D_n_^−3^* (Equation (3)). However, in the last part of the polymerizations, the values of *N_p_* for both polymerizations show a slight decrease, which is more evident in the polymerization at the lower dosing rate in such way that the final *N_p_* values are higher for the higher dosing rate. This fact suggests the occurrence of a certain degree of particle coalescence at the end of the polymerizations.

### 2.3. Gel Percentage

**Figure 5:**
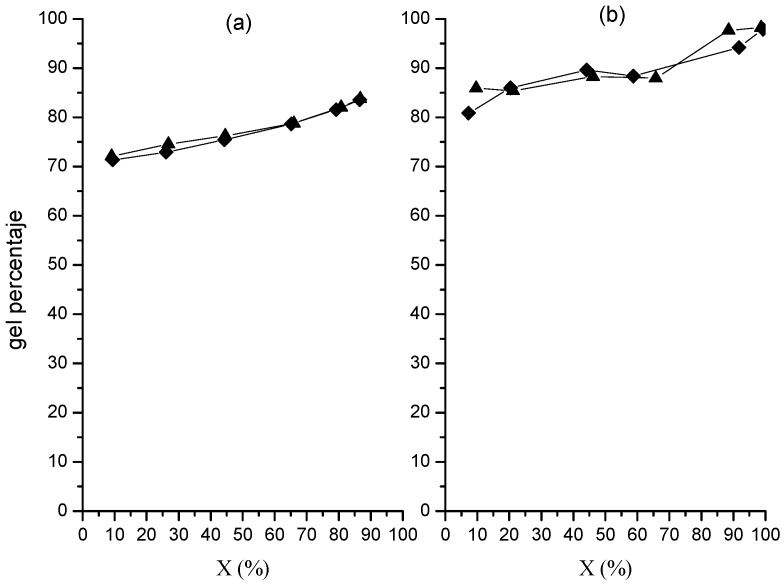
Variation of gel percentage in polymer in nanoparticles with global conversion from polymerizations at 0.05 (**a**) and 0.15 (**b**) g/min in organic phase dosing rate. Runs M (♦); runs MR (▲).

Figure 5 depicts how the gel percentage of polymer in the nanoparticles evolves with the polymerization, showing a continued increase for both cases. Gel percentage is defined as the polymer fraction which turns non-solvent soluble as a result of the cross-linking between polymer chains.

Here, the larger values correspond to the run at higher dosing rate, with 80%–85% at the initial part of the polymerization and around 98% at the end of it. In contrast, the other polymerization shows values ranging 70% to 84% in the same interval. The continued increase in gel percentage could be explained as a result of an increase in the chain number inside the particles as the polymerization progresses, which would increase the number of cross-linking points. Furthermore, the direct dependence of the gel percentage on the dosing rate would also arise from a possible increase in the polymer concentration in the particles.

### 2.4. Porosity and Pore Diameter

**Figure 6:**
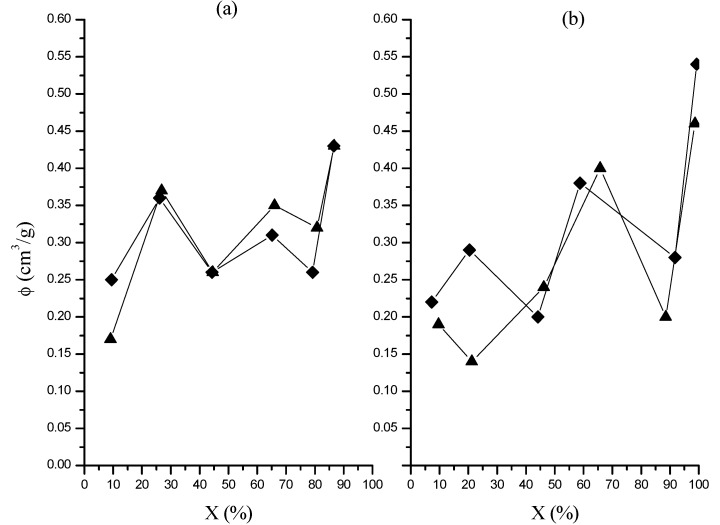
Evolution of nanoparticles porosity with global conversion in polymerizations at 0.05 (**a**) and 0.15 (**b**) g/min in organic phase dosing rate. Runs M (♦); runs MR (▲).

**Figure 7:**
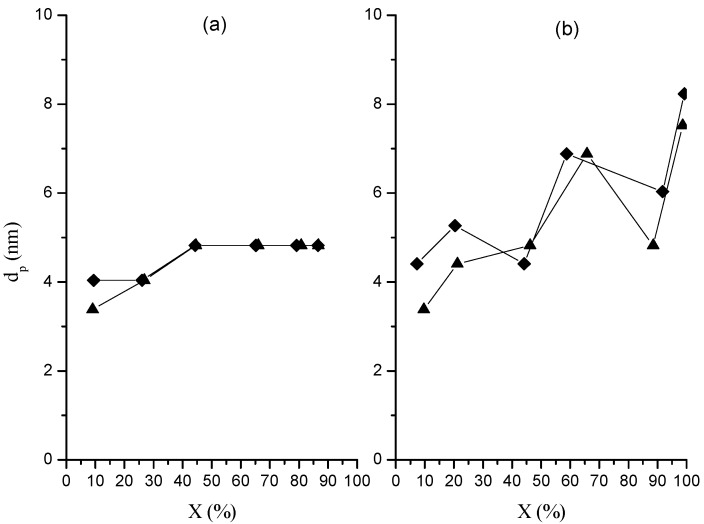
Change of pore diameter in nanoparticles with global conversion in polymerizations at 0.05 (**a**) and 0.15 (**b**) g/min in organic phase dosing rate. Runs M (♦); runs MR (▲).

Porosity and average pore diameter (*d_p_*) values as a function of global conversion are shown in Figure 6 and Figure 7, respectively.

Irrespective of the dosing rate both variables show a continued increase with conversion, with slightly larger values in porosity (*ca.* 0.5 cm^3^/g) and *d_p_* (8 nm) for the polymerization at higher dosing rate. The relatively low porosity and pore diameters values obtained in this study were similar to those obtained in our previous work in which similar polymerization conditions were employed [13]. On the other hand, the porosity increase with conversion could be explained as a result of the increase in the gel percentage also as the polymerization evolves. This would cause that toluene not be absorbed by the cross-linked chains, which would lead to a phase separation (macrosyneresis) with the continuous phase composed of toluene, unreacted monomers and the dissolved polymer [23]. Furthermore, as the cross-linking increases the chains form compact nanospheres whose number rises with global conversion, resulting in an increment in the number of voids between nanospheres and finally, in an increase in the porosity after toluene evaporation.

### 2.5. Surface Tension

**Figure 8:**
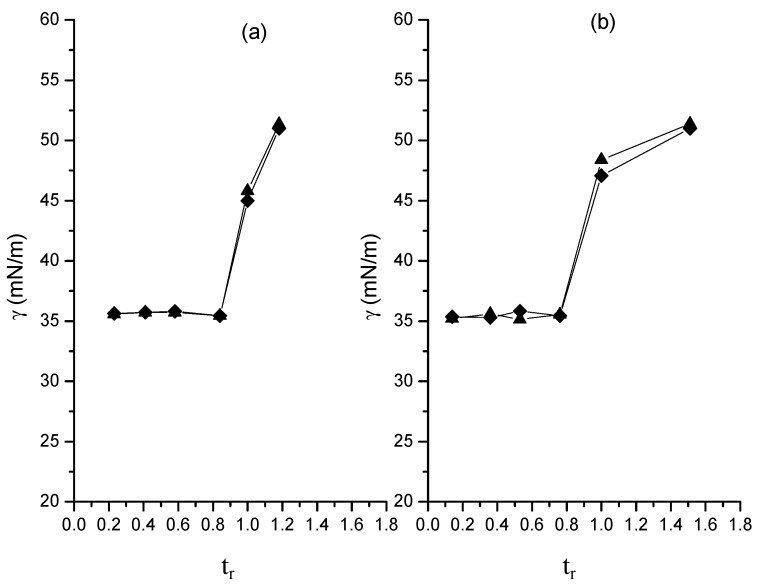
Surface tension of latexes as a function of relative time in polymerizations at 0.05 (**a**) and 0.15 (**b**) g/min in organic dosing rate. Runs M (♦); runs MR (▲).

Figure 8 shows the variation of latex surface tension (γ) with *t_r_*. The behavior of γ in both polymerizations is similar, that is, values close to 35 mN/m up to *t_r_* ≈ 0.8, followed by an increase up to ≈ 45 mN/m at *t_r_* = 1, attaining a value close to 55 mN/m at the end of the polymerization.

The γ value close to 35 mN/m suggests the presence of micelles [24] up to at least *t_r_* ≈ 0.8, while the higher values indicate that micelles have disappeared and all surfactant in the latex is on the nanoparticles’ surface. In accordance with Figure 3, particle nucleation occurs up to approximately 80% in global conversion for the polymerizations at lower dosing rate and up to 90%, for the other one. Undoubtedly, the presence of micelles up to at least *t_r_* ≈ 0.8, which corresponds to 60%–65% global conversion, is the determinant factor for the particle formation. Therefore, it would be interesting to know why particle formation occurs at higher conversions. In fact, according to Figure 8, the disappearance of micelles not necessarily takes place at *t_r_* ≈ 0.8 for both polymerizations, but this event occurs in a point between *t_r_*≈ 0.8 and *t_r_* = 1, so most probably the micelles are present up to near *t_r_* = 1, allowing the particle formation up to that point. To explain why particle coalescence occurs at the end of the polymerizations the surface coverage ratio, *r*, defined as the total surface of particles that the available surfactant would cover forming a saturated monolayer divided by the total surface area of the particles at *t_r_* = 1was calculated. For this task data from the recipe and the values of *D_n_*, *N_p_*, *X*, density of the porous particle and the area per SDS molecule in a saturated monolayer on particle surface (0.5 nm^2^/molecule) [25] were used. The *r* values for M5 and M5R were 0.56 and 0.54, respectively, for the polymerizations carried out at the higher dosing rate, while the polymerizations at the lower dosing rate showed the same value of 0.55 for M5 and M5R runs. The magnitude of these *r* values indicates that the particles are covered by a layer of surfactant with approximately half of the number of molecules required for saturation. This implies a certain degree of particle instability, which would result in the particle coalescence observed at the end of the polymerization. The low *r* values and the fact that they are practically the same for both dosing rates could also explain why a smaller particle size at the lower dosing rate was not achieved. What probably occurs is that a value close to 0.55 is the lowest value that *r* can achieve under the conditions at which the polymerizations were carried out and since smaller particle sizes require lower *r* values, an average particle close to 28 nm is the minimum particle size that can be attained. In fact, the *r* values for the particles obtained at the end of the polymerizations increase up to 0.6–0.7, which would result from particles that coalesce trying to reduce the area to volume ratio and thereby increase their stability.

### 2.6. Monomer Concentrations in Particles

**Figure 9:**
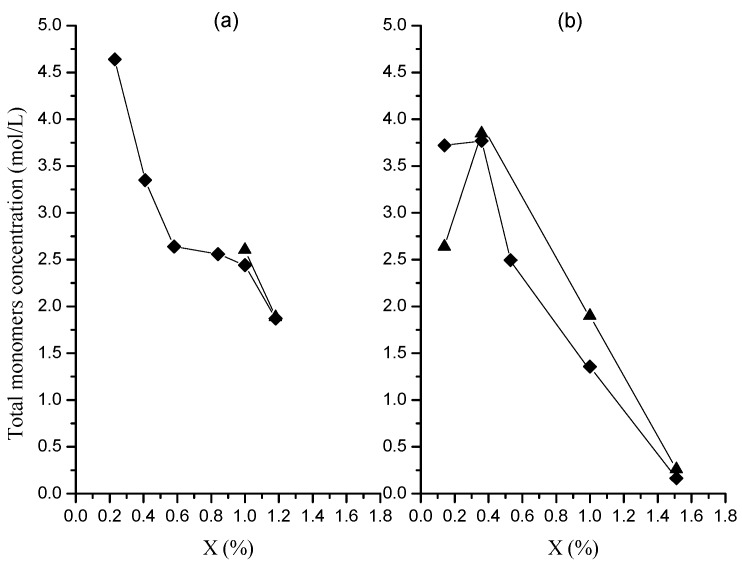
Evolution of total monomers concentration inside nanoparticles with global conversion in polymerizations at 0.05 (**a**) and 0.15 (**b**) g/min in organic dosing rate. Runs M (♦); runs MR (▲).

St and DVB concentrations in the particles along the course of the polymerizations were calculated as follows: from the recipe, phase-organic dosing rate and conversion data at a given time, the amounts of toluene, St and DVB in the latex were known. The analysis by GC gave the St/DVB weight ratio in the particles, which with the amounts mentioned above, allowed to know the composition of the organic phase in the latex. Then, the assumption that the composition of the mixture of toluene, St and DVB swelling particles and micelles was the same was made. The partitioning of this mixture between particles and micelles was estimated from the polymer/surfactant weight ratio, since this datum, along that of the total surfactant in the recipe, allowed to know the surfactant available to form micelles and from here the number of micelles and its content of organic phase. In this calculation an aggregation number of 60 [26] and a molecular surface coverage for SDS micelles of 0.5 nm^2^/molecule [25] were taken. With the partitioning results, the particle composition was known and St and DVB concentrations were calculated under the assumption of additive volumes of the components and discarding the volume of the cross-linked polymer from the total volume available for carrying out the propagation reactions. Table 1 and Table 2 summarize the results achieved and Figure 9 shows the variation of total monomers concentration in the particles during the polymerizations.

**Table 1:** Most important data used in the estimation of monomer concentration in particles.

Organic Phase Dosing Rate (g/min)	*t_r_*	Sulfur Content in Polymer-Surfactant Mixture (%)		Available Surfactant in the Latex for Forming Micelles (g)		Toluene and Residual Monomer in Micelles (g)		Residual Monomers in Particles(g)				Total Polymer in Particles (g)		Total Non-Cross-Linked Polymer in Particles (g)	
								St		DVB					
0.05		**M**	**MR**	**M**	**MR**	**M**	**MR**	**M**	**MR**	**M**	**MR**	**M**	**MR**	**M**	**MR**
	0.23	3.71	NA	3.42	NA	1.64	NA	2.04	NA	0.32	NA	1.86	2.48	0.66	0.66
	0.41	2.79	NA	2.08	NA	0.99	NA	2.84	NA	0.19	NA	5.15	5.38	1.80	1.67
	0.58	2.65	NA	0.88	NA	0.41	NA	2.84	NA	0.34	NA	11.70	12.20	2.72	2.61
	0.84	1.88	NA	1.00	NA	0.47	NA	3.89	NA	0.55	NA	15.02	16.39	3.55	3.48
	1.00	NR	NR	0.00	0.00	0.00	0.00	4.43	4.78	0.47	0.53	23.22	22.60	3.80	3.63
	1.18	NR	NR	0.00	0.00	0.00	0.00	3.11	3.10	0.27	0.53	25.54	25.04	3.63	3.58
0.15	0.14	4.22	3.90	3.20	2.97	1.53	1.41	0.44	0.20	0.33	0.17	2.29	2.37	0.36	0.35
	0.36	3.92	3.96	1.58	1.33	0.76	0.64	1.02	1.69	2.49	1.17	6.66	6.55	0.72	0.79
	0.53	2.71	NA	0.55	NA	0.26	NA	2.17	NA	0.20	NA	11.09	10.97	1.21	1.43
	0.76	NA	NA	NA	NA	NA	NA	NA	NA	NA	NA	16.64	16.37	1.75	1.97
	1.00	NR	NR	0.00	0.00	0.00	0.00	1.26	1.67	0.83	1.25	20.58	20.19	1.35	0.52
	1.51	NR	NR	0.00	0.00	0.00	0.00	0.09	0.13	0.11	0.19	22.10	22.06	0.54	0.44

NA-not available; NR-not required, since from γ values it is known that all the surfactant is on the particles.

**Table 2:** Monomer concentration in particles along polymerizations as function of organic phase dosing rate.

0.05 g/min					0.15 g/min				
*t_r_*	St (mol/L)		DVB (mol/L)		*t_r_*	St (mol/L)		DVB (mol/L)	
	M	MR	M	MR		M	MR	M	MR
0.23	4.12	NA	0.52	NA	0.14	2.32	1.56	1.40	1.08
0.41	3.18	NA	0.17	NA	0.36	1.28	2.14	2.49	1.71
0.58	2.41	NA	0.23	NA	0.53	2.32	NA	0.17	NA
0.84	2.30	NA	0.26	NA	0.76	NA	NA	NA	NA
1.00	2.25	2.39	0.19	0.21	1.00	0.89	1.19	0.47	0.71
1.18	1.74	1.74	0.12	0.15	1.51	0.09	0.12	0.08	0.14

NA-not available.

The data in Table 1 and Table 2 show that St concentrations in the particles are consistently higher for the polymerization at both dosing rates. Consequently, the total monomer concentration (Figure 9) is higher for the polymerization at the lower dosing rate practically along all the reaction. This behavior is somewhat expected due to the higher *x_i_* values obtained at the polymerization carried out at the higher dosing rate.

### 2.7. Polymerization Rate and Average Number of Radicals per Particle

**Figure 10:**
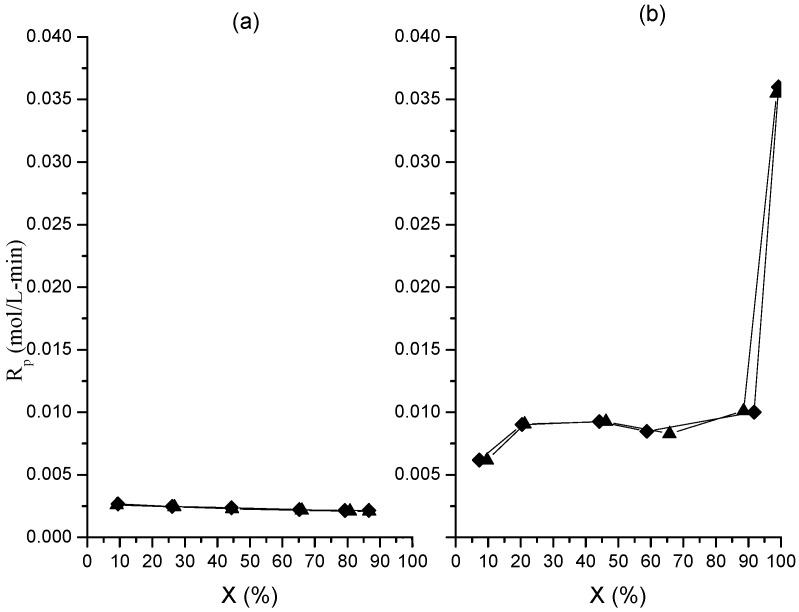
Evolution of polymerization rate with global conversion in polymerizations at 0.05 (**a**) and 0.15 (**b**) g/min in organic phase dosing rate. Runs M (♦); runs MR (▲).

The variation of the polymerization rate (*R_p_*) and the average number of radicals per particle (*ñ*) as the polymerization evolves is shown in Figure 10 and Table 3, respectively. The *R_p_* values (in mol/L water-min) were obtained by fitting *X vs. t* data to a polynomial equation and evaluating its derivative at some given points along the polymerization using the recipe data. On the other hand, *ñ* values were calculated from the well-known equation representing the polymerization rate in an emulsion polymerization and which applies in our case:

(5) $$R_{p}=\frac{k_{p}\left[M_{p}\right]N_{p}\text{ñ}}{N_{A}}$$

where *k_p_* is the propagation rate constant and *N_A_*, the Avogadro’s number.

Given that this equation would be used in a copolimerization where St and DVB are monomer 1 and 2, respectively, the terms *[M]_p_* and *k_p_* were redefined, obtaining:

(6) $$R_{p}=\;\left(k_{p11}\left[St\right]+k_{p22}\left[DVB\right]+k_{p12}\left[DVB\right]+k_{p21}\left[St\right]\right)\frac{\text{ñ}N_{p}}{N_{A}}$$

where *k*_11_ and *k*_22_ are the homo-propagation rate constants for St and DVB, respectively and *k*_12_ and *k*_21_, the corresponding cross-propagation rate constants. Despite the fact that DVB is a mixture of *m*-DVB and *p*-DVB, to simplify the calculations it was assumed that DVB is only composed of *m*-DVB. The values taken from literature [27] for *k*_11_, *k*_22_, *k*_12_ and *k*_21_ are 480, 456, 1140 and 456 L·mol^−1^·s^−1^, respectively. Once all the constants and the rest of the variables in Equation (6) were known, *ñ* was calculated at different points along the polymerization.

**Table 3:** Evolution of *ñ* values along polymerizations.

0.05 g/min			0.15 g/min		
*t_r_*	*Ñ* × 10^4^		*t_r_*	*ñ* × 10^3^	
	M	MR		M	MR
0.23	6.9	NA	0.14	3.3	3.7
0.41	6.0	NA	0.36	1.8	2.3
0.58	3.4	NA	0.53	1.7	NA
0.84	2.6	NA	0.76	NA	NA
1.00	2.7	2.3	1.00	1.8	1.2
1.18	4.4	3.8	1.51	47.7	33.8

Figure 10 shows that *R_p_* increases at the beginning of the polymerization, remaining almost constant from approximately 20% up to 90% global conversion, with a pronounced increase at the end of the reaction for the polymerization at the higher dosing rate. In contrast, the *R_p_* values at the lower dosing rate are significantly lower and show a slightly decrease as polymerizations evolve. On the other hand, Table 3 reveals that *ñ* behavior is similar for both polymerizations, that is to say, a decrease from the beginning to the end of the organic phase dosing, followed by an increase at the end of the polymerization. However, the *ñ* values for the polymerization at higher dosing rate are significantly higher; furthermore, the final increase is also more marked, attaining values around to 3.4 × 10^−2^–4.8 × 10^−2^ radicals per particle against 3.8 × 10^−4^–4.4× 10^−4^ for the polymerization at the other dosing rate.

The above results suggest that the direct dependence of *x_i_* on the organic-phase dosing rate arises from the higher values of *ñ* in the polymerization at the higher dosing rate, which would lead to an increase in the monomer consumption. This supports the previous explanation given to understand the direct dependence of gel percentage on dosing rate (Figure 5), which said that the probability of cross-linking reactions occurrence was increased due to the increase of the propagation reactions occurrence inside the particles.

The question now emerging is why *ñ* increases are so high with the increase in the organic-phase dosing rate. The probable cause would be that proposed by Capek [28], who stated that the cross-linked polymer chains would trap the propagating radicals. It is believed that these chains would act as barriers preventing two radicals encounter, thereby retarding the bimolecular termination reactions and consequently, extending the life span of the radicals inside the particles.

## 3. Experimental Section

### 3.1. Reagents

St (99%), DVB (99%), sodium dodecylsulfate (SDS) (98.5%) and ammonium persulfate (APS) were from Sigma-Aldrich (Toluca, Edo. Méx., México) Monomers and toluene (99.9%), which were obtained from J.T. Baker (Monterrey, N.L., México), were distilled under reduced pressure and stored at 4 °C. SDS and APS were used as received. Deionized and triple-distilled water was drawn from a Millipore system.

### 3.2. Polymerizations

The reactions were carried out in a 150 mL jacketed glass reactor equipped with a reflux condenser and mechanical agitation in accordance with the following procedure: the required quantities of water, SDS and APS were charged into the reactor, subjected to 650 rpm agitation and bubbled with argon for 1 h, after which the temperature of the solution was raised to 70 °C and the addition of the organic phase composed of toluene and monomers started at a pre-determined dosing rate using a KD Scientific from Sigma-Aldrich syringe pump. After the end of the organic phase addition the reaction was allowed to proceed for a given time (post-addition period). Due to the large sample amounts required for all characterizations a series of runs were carried out for the two organic phase dosing rates (*F_o_*) studied, that is to say, 0.05 and 0.15 g/min. For a given *F_o_*, the addition period in the polymerization was divided in 5 intervals and the complete polymerization was considered as the interval 6. Then a run (in duplicate) for each interval was carried out, stopping the polymerization and collecting all the latex at the end of each of them. For 0.05 g/min polymerization the intervals 1, 2, 3, 4 and 5 included from the beginning of organic phase addition to *t_r_* equal to 0.23, 0.41, 0.58, 0.84 and 1, respectively. For 0.15 g/min polymerization, *t_r_* values for the same intervals were 0.14, 0.36, 0.53, 0.76 and 1.This way, the amount of latex obtained was enough to make all the required characterizations.

**Table 4:** Formulations used in the polymerizations at the organic-phase dosing rate of 0.05 g/min.

	M1	M2	M3	M4	M5	M6	M1R	M2R	M3R	M4R	M5R	M6R
Water (g)	90.68	90.65	90.90	90.51	90.58	90.35	90.40	90.60	90.51	90.74	90.66	90.83
SDS (g)	4.75	4.50	4.54	4.58	4.53	4.60	4.50	4.50	4.51	4.57	4.59	4.63
APS (g)	0.04	0.04	0.04	0.04	0.04	0.04	0.04	0.04	0.04	0.04	0.04	0.04
Organic Phase (g)	7.60	13.70	19.20	28.40	34.00	34.00	7.70	13.70	19.10	28.30	34.00	34.00
Organic Phase Composition												
Toluene (wt. %)	24.92	24.89	24.68	25.02	25.34	24.50	24.95	24.59	25.00	24.94	24.72	24.96
St (wt. %)	60.04	60.37	60.43	59.95	59.65	60.63	60.13	60.48	59.95	59.79	60.44	60.06
DVB (wt. %)	15.04	14.74	14.89	15.03	15.01	14.87	14.93	14.93	15.05	15.27	14.84	14.98

**Table 5:** Formulations used in the polymerizations at the organic-phase dosing rate of 0.15 g/min.

	M1	M2	M3	M4	M5	M6	M1R	M2R	M3R	M4R	M5R	M6R
Water (g)	90.50	90.51	90.96	90.51	90.50	90.59	90.40	90.60	90.51	90.74	90.66	90.83
SDS (g)	4.52	4.58	4.51	4.51	4.50	4.57	4.50	4.50	4.51	4.57	4.59	4.63
APS (g)	0.04	0.04	0.04	0.04	0.04	0.04	0.04	0.04	0.04	0.04	0.04	0.04
Organic Phase (g)	4.70	12.10	18.80	25.80	33.90	34.10	4.69	12.10	18.80	25.10	33.90	33.80
Organic Phase Composition												
Toluene (wt. %)	24.92	24.89	24.68	25.02	25.34	24.50	24.95	24.59	25.00	24.94	24.72	24.96
St (wt. %)	60.04	60.37	60.43	59.95	59.65	60.63	60.13	60.48	59.95	59.79	60.44	60.06
DVB (wt. %)	15.04	14.74	14.89	15.03	15.01	14.87	14.93	14.93	15.05	15.27	14.84	14.98

Table 4 and Table 5 include the formulation for all the runs carried out in this study. For both *F_o_* studied, the runs stopped at the end of the intervals 1, 2, 3, 4 and 5 were named as M1, M2, M3, M4 and M5, respectively. The run that includes the complete polymerization was named as M6. The suffix R was used to identify the replicates of the runs.

### 3.3. Characterization

#### 3.3.1. Particle Size

Determinations of particle size distributions for latexes samples were carried out in a JSM-7401F scanning-transmission electron microscope (STEM, JEOL, México City, México). For the measurements a dilution containing about 2.5 g polymer per liter was prepared, one drop of it was deposited on a copper grid and allowed to dry. The diameters of a number of particles ranging from 500 to 1000 were measured from the set of micrographs to obtain the number-average diameter (*D_n_*), through the following equation:

(7) $$D_{n}=\frac{\sum_{i}D_{i}}{\sum_{i}n_{i}}=\frac{\sum n_{i}D_{i}}{n}$$

#### 3.3.2. Gel Percentage

First, the surfactant was removed from the latex samples by dialysis using porous membranes from Sigma-Aldrich with molar masses larger than 12,000 g/mol in exclusion size. Dialysis was carried out until the electrical conductivity values of the fresh and recovered water used in the daily operation were similar. Then surfactant-free dried polymer (0.05 g) wasplaced in a test tube containing toluene (2.5 g), which was sealed and allowed to stand for 48 h. After that, the mixture was centrifuged for 1 h at room temperature in an Optima XI-100K apparatus (Instrumentos y Equipos Falcón, Monterrey, N.L., México) at 95,000 rpm; then, the supernatant liquid was discarded and the solids was dried to constant weight at 90 °C. The gel content was calculated from the ratio of the weight of the dried solids from centrifugation to that of the original dried polymer.

#### 3.3.3. Porosity and Pore Size

Porosity (ϕ) and pore diameter (*d_p_*) of dried particles from latexes were determined in Quantachrome Autosorb 1 MPR automated surface area and pore size analyzer (CISASA, León, Gto., México) using the Quantachrome software and the density fluctuation theory (DFT) [29].

#### 3.3.4. Surface Tension

This latex property were measured at 25 °C in a Wilhelmy plate tensiometer Sigma 703 from KVS Instruments from Sigma-Aldrich.

#### 3.3.5. Sulfur Content in the Polymer-Surfactant Mixture

The dried solids recovered from the latex were analyzed in an Eltra CS800 induction furnace (Tecanalitic, Saltillo, Coah., México) by the combustion method to determine the sulfur content. Only the samples of those latexes containing micelles were analyzed, for which the results of surface tension measurements were considered. With the data of sulfur content and the molecular weight of the SDS the surfactant/polymer weight ratio was calculated and from here, the total surfactant stabilizing the particles was known.

#### 3.3.6. Styrene/Divinylbenzene Ratio in Particles

The St/DVB weight ratio in the particles along the polymerization was estimated from the particle analysis in a HP6890 gas chromatograph (Agillent Technologies, México City, México) with a FID detector and a G1888 headspace sampler. To prepare the samples, latex (5 g) was centrifuged for 1 h at room temperature in an Optima XI-100K apparatus at 95,000 rpm, after which the particles were recovered and subjected to analysis by gas chromatography (GC). To know the content of St and DVB in the particles the calibration curves for both of monomers were previously plotted.

## 4. Conclusions

Semicontinuous heterophase polymerization carried out under monomer starved conditions allowed us to prepare latexes containing mesoporous polystyrene nanoparticles with a relatively high solids content (≈20%) and all the formulation surfactant on the particles. The unexpected direct relationship between monomer dosing rate and instantaneous conversion was found to be the major result in this study, however, the absence of particle size dependence on monomer dosing rate also was notable. It was postulated that the reason for the former finding was the higher *ñ* values attained in the polymerization at the higher dosing rate; this would arise from the higher fraction of cross-linked polymer obtained, whose chains would trap the radicals retarding the bimolecular termination reactions and allowing the existence of more than one radical inside the particles, which in turn would increase the propagation rate. The absence of monomers dosing rate effect on particle size was explained as possibly resulting from the achievement of the minimum particle size that the surfactant in the formulation can stabilize under the set of conditions at which the polymerizations were carried out.

## References

[1] Arshady R., Lewith A. (1983). Suspension polymerization and its application to the preparation of polymers supports. React. Polym..

[2] Maillard-Terrier M.C., Cazé C. (1984). Texture pore use de copolymères 4-vinylpyridine divinylbenzène. Eur. Polym. J..

[3] Arshady R. (1991). Beaded polymer supports and gels. II. Physico-chemical criteria and functionalization. J. Chromatogr. A.

[4] Chengyou K., Huihui L., Qing Y., Yiangzheng K. (1997). Preparation of porous latex particles by emulsion polymerization. Korea Polym..

[5] Liu Q., Li Y., Shen S., Shanshan Z. (2011). The influence of crosslinking density on the pore morphology of copolymer beads prepared with a novel pore-forming agent. Mater. Chem. Phy..

[6] Liu Q., Duan Y., Zhou Z., Wang J., Wang M., Shen S. (2012). The influence of different porogens with halogen substituents on the pore structure of polydivinylbenzene beads. Mater. Chem. Phy..

[7] Zhu H., Chen H., Zeng X., Wang Z., Zhang X., Wu Y., Gao Y., Zhang J., Liu K. (2014). Co-delivery of chemoterapeutic drugs with vitamin E TPGS by porous PLGA nanoparticles for enhanced chemoteraphy against multi-drug resistance. Biomaterials.

[8] Kowalczuk A., Trzcinska R., Trzebicka B., Muller A.H.E., Dworak A., Tsvetanov C.B. (2014). Loading of polymer nanocarriers: Factors, mechanisms and applications. Prog. Polym. Sci..

[9] Gu X., Sun Z., Wu S., Qi W., Wang H., Xu X., Su D. (2013). Surfactant-free hydrothermal synthesis of sub-10 nm γ-Fe_2_O_3_-polymer porous composites with high catalytic activity for reduction of nitroarenes. Chem.Commun..

[10] Xie Y.M., Lu L., Li M.H., Pan B.C., Chen Q., Zhang W.M., Zhang Q.X. (2012). Development of cation exchanger-based nano-CdS hybrid catalyst for visible-light photodegradation of rhodamine B from water. Sci. China Chem..

[11] Wang X., Zhou Z., Jing G. (2013). Synthesis of Fe_3_O_4_ poly(styrene-glycidyl methacrylate) magnetic porous microspheres and application in the immobilization of *Klebsiella* sp. FD-3 to reduce Fe(III)EDTA in a NO(x) scrubbing solution. Bioresour. Technol..

[12] Lee J.-Y., Kim J.-H. (2004). Highly porous organic nanoparticles formed from supercritical carbon dioxide mediated sol-emulsion-gel method. Chem. Lett..

[13] Esquivel O., Treviño M.E., Saade H., Puig J.E., Mendizábal E., López R.G. (2011). Mesoporous polystyrene nanoparticles synthesized by semicontinuous heterophase polymerization. Polym. Bull..

[14] Wu X., Li H., Xu Y., Xu B., Tong H., Wang L. (2014). Thin film fabricated from solution-dispersable porous hyperbranched conjugated polymer nanoparticles without surfactants. Nanoscale.

[15] Thassu D., Deleers M., Pathak Y. (2007). Nanoparticulate Drug Delivery System.

[16] Ledezma R., Treviño M.E., Elizalde L.E., Pérez-Carrillo L.A., Mendizábal E., Puig J.E., López R.G. (2007). Semicontinuous heterophase polymerization under monomer starved conditions to prepare nanoparticles with narrow size distributions. J. Polym. Sci. A Polym. Chem..

[17] Aguilar J., Arellano M.R., Rabelero M., Nuño-Donlucas S.M., Mendizábal E., López R.G., Puig J.E. (2011). Narrow size-distribution nanoparticles of poly(methyl methacrylate) made by semicontinuous heterophase polymerization. J. Appl. Polym. Sci..

[18] Pérez-García M.G., Rabelero M., Nuño-Donlucas S.M., Mendizábal E., Martínez-Richa A., López R.G., Arellano M., Puig J.E. (2012). Semicontinuous heterophase polymerization of *n*-butyl methacrylate: Effect of monomer feeding rate. J. Macromol. Sci. Pure Appl. Chem..

[19] Krackeler J.J., Naidus H. (1969). Particle size and molecular weight distributions of various polystyrene emulsions. J. Polym. Sci. Part. C.

[20] Smith W.V., Ewart R.H. (1948). Kinetics of emulsion polymerization. J. Chem. Phys..

[21] Puig J.E., Mendizábal E., López-Serrano F., López R.G., Somasundaran P. (2012). Surfactant assisted polymerization methods. Encyclopedia of Surface and Colloids Science.

[22] Schrader D., Brandrup J., Immergut E.H., Grulke E.A. (1999). Physical constants of some important polymers. Polymer Handbook.

[23] Dusek K., Chompff A.J., Newman S. (1971). Inhomogeneities induced by crosslinking in the course of crosslinking copolymerization. Polymer Networks: Structure and Mechanical Properties.

[24] Sajjadi S., Brooks B.W. (2000). Unseeded semibatch emulsion polymerization of butyl acrylate: Bimodal particle size distribution. J. Polym. Sci. A Polym. Chem..

[25] Ramírez A.G., López R. G., Tauer K. (2004). Studies on semibatch microemulsion polymerization of butyl acrylate: Influence of the potassium peroxidisulfate concentration. Macromolecules.

[26] Gehlen M.H., de Schryver F.C. (1993). Fluorescence quenching in micelles in the presence of a probe-quencher ground-state charge-transfer complex. J. Phys. Chem..

[27] Vivaldo-Lima E. (1998). Development of an Effective Model for Particle Size Distribution in Suspension Copolimerization of Styrene/Divinilbenzene. Ph.D. Thesis.

[28] Capek I. (1996). On the kinetics of heterogeneous free radical crosslinking polymerization. J. Dispers. Sci. Technol..

[29] Duda J.T., Jagiello L., Jagiello J., Milewska-Duda J. (2007). Complementary study of microporous adsorbents with DFT and LBET. Appl. Surf. Sci..

